# Скрининг акромегалии среди пациентов с гиперпролактинемией и аденомой гипофиза

**DOI:** 10.14341/probl13344

**Published:** 2023-09-18

**Authors:** Ю. А. Уханова, И. А. Иловайская

**Affiliations:** Московский областной научно-исследовательский клинический институт им. М.Ф. Владимирского; Московский областной научно-исследовательский клинический институт им. М.Ф. Владимирского

**Keywords:** инсулиноподобный фактор роста 1, акромегалия, гиперпролактинемия, аденома гипофиза, скрининг

## Abstract

**ОБОСНОВАНИЕ:**

ОБОСНОВАНИЕ. Гиперпролактинемия является распространенным эндокринным расстройством и примерно в 25–39% случаев сопровождает избыток секреции гормона роста. В российских клинических рекомендациях при выявлении акромегалии рекомендовано определение уровня пролактина. Однако нет четкого понимания необходимости исследования уровня ИРФ-1 (инсулиноподобный ростовой фактор 1 типа) у пациентов с подтвержденной патологической гиперпролактинемией и опухолью гипофиза.

**ЦЕЛЬ:**

ЦЕЛЬ. Определить частоту исследования уровня ИРФ-1 у пациентов с гиперпролактинемией и аденомой гипофиза в условиях реальной клинической практики, а также выявить долю пациентов с акромегалией среди данной когорты.

**МАТЕРИАЛЫ И МЕТОДЫ:**

МАТЕРИАЛЫ И МЕТОДЫ. В период с декабря 2019-го по декабрь 2022 гг. проведено одноцентровое обсервационное одномоментное одновыборочное неконтролируемое исследование. На его первом этапе, по данным медицинских карт, путем сплошного включения наблюдений определена доля пациентов с аденомой гипофиза и гиперпролактинемией, у которых исследовался уровень ИРФ-1. На втором этапе исследования доступным для наблюдения пациентам без известных показателей ИРФ-1 проведено его определение, в случае повышения уровня ИРФ-1 проведено исследование концентрации СТГ (соматотропного гормона) в ходе теста с нагрузкой глюкозой.

**РЕЗУЛЬТАТЫ:**

РЕЗУЛЬТАТЫ. На первом этапе в исследование было включено 105 пациентов, из них уровень ИРФ-1 был определен в 41/105 (39%) случае. Среди них в подгруппе с инциденталомами гипофиза — в 22/41 (53,7%) случаях, в подгруппе с гиперпролактинемией — в 19/64 (29,7%) случаях. На втором этапе дополнительно уровень ИРФ-1 определен еще у 53 пациентов с гиперпролактинемией и аденомой гипофиза (всего 94 пациента). Уровень ИРФ-1 был повышен у 11/94 пациентов, в дальнейшем акромегалия подтверждена у 3/94 пациентов (3,2%).

**ЗАКЛЮЧЕНИЕ:**

ЗАКЛЮЧЕНИЕ. В реальной клинической практике у пациентов с аденомой гипофиза и гиперпролактинемией уровень ИРФ-1 исследуется лишь в 39% случаев. При активном скрининге из 94 человек с гиперпролактинемией и аденомой гипофиза, у которых не было характерных клинических проявлений акромегалии, заболевание удалось выявить в 3 случаях (3,2%). Мы считаем исследование уровня ИРФ-1 в качестве скрининга акромегалии у пациентов с гиперпролактинемией и аденомой гипофиза оправданным.

## Abstract (en)

**BACKGROUND:**

BACKGROUND: Hyperprolactinemia accompanies growth hormone hypersecretion in approximately 25-39% of cases. There is a recommendation to determine the level of prolactin in clinical guidelines for diagnosis and treatment of acromegaly. However, there is no understanding of the necessity to investigate the IGF-1 level in patients with hyperprolactinemia and a pituitary adenoma.

**AIM:**

AIM: Determining the proportion of patients with hyperprolactinemia and pituitary adenoma, who were examined for IGF-1 levels, and identifying the proportion of patients with acromegaly among this cohort.

**MATERIALS AND METHODS:**

MATERIALS AND METHODS: Between December 2019 and December 2022 a single-center observational single-stage single-sample uncontrolled study was conducted. At the first stage of the study, the proportion of patients with pituitary adenoma and hyperprolactinemia with studied IGF-1 levels was determined, according to medical records. At the second stage of the study, patients without known indicators of IGF-1 were determined. The concentration of growth hormone was studied during the oral glucose load in the case of increased IGF-1 levels.

**RESULTS:**

RESULTS: At the first stage, 105 patients were included in the study. The level of IGF-1 was determined in 41/105 (39%) cases. There were 22/41 (53.7%) cases in the subgroup with pituitary incidentalomas and 19/64 (29.7%) cases in the subgroup with hyperprolactinemia among them. At the second stage, the IGF-1 level was additionally determined in 53 patients with hyperprolactinemia and pituitary adenoma (total 94 patients). The level of IGF-1 was elevated in 11/94 patients, further acromegaly was confirmed in 3/94 patients (3.2%).

**CONCLUSION:**

CONCLUSION: In real clinical practice the level of IGF-1 is studied only in 39% of cases in patients with pituitary adenoma and hyperprolactinemia. The disease was detected in 3 cases (3.2%) out of 94 people with hyperprolactinemia and pituitary adenoma without clinical manifestations of acromegaly. We consider the study of IGF-1 levels justified as a screening for acromegaly in patients with hyperprolactinemia and pituitary adenoma.

## ОБОСНОВАНИЕ

Акромегалия длительное время может скрываться под масками разных заболеваний, которые впоследствии могут оказаться ее осложнениями. Согласно анализу данных 19 национальных регистров акромегалии, от первых клинических признаков до постановки диагноза проходит от 5 до 14 лет [1]. По данным единого российского регистра опухолей гипоталамо-гипофизарной области, у пациентов с акромегалией средний период времени от появления первых симптомов заболевания до постановки диагноза составляет 3 года [2]. Но наш собственный клинический опыт показывает, что при оценке длительности акромегалии врачи часто опираются на появление изменений внешности, не обращая внимания на длительность артериальной гипертензии, сахарного диабета, стоматологических проблем и других возможных ее косвенных проявлений. Поэтому реальная длительность гиперсекреции гормона роста может быть больше.

Распространенными осложнениями акромегалии являются заболевания сердечно-сосудистой системы, нарушения углеводного обмена, синдром обструктивного апноэ сна, заболевания опорно-двигательного аппарата, онкологические заболевания, синдром запястного канала, которые сами по себе являются распространенными и значимыми для пациентов [3–7]. Заподозрить избыток секреции гормона роста зачастую удается, только когда уже формируется комплекс вышеперечисленных заболеваний, так как первые проявления акромегалии могут быть неспецифичны. С другой стороны, сокращение длительности активной стадии акромегалии приводит к снижению количества ассоциированных заболеваний [8]. Поэтому скрининг акромегалии набирает популярность во всем мире [9][10].

Danilowicz K., et al. в своей статье представили национальную программу скрининга акромегалии в Латинской Америке, обосновали ее необходимость и осветили условия реализации [11]. Таким образом, проблема поиска фокусных групп пациентов для скрининга акромегалии остается актуальной.

В 2011 г. Rosario P.W., et al. провели скрининг акромегалии среди пациентов с сахарным диабетом и нарушением толерантности к глюкозе, по результатам которого из 2270 человек удалось диагностировать 3 случая акромегалии [12]. В 2012 г. были опубликованы результаты скрининга, проведенного на основании опросника среди пациентов учреждений первичной медико-санитарной помощи на предмет увеличения конечностей. Из 178 человек, ответивших положительно как минимум на один из заданных вопросов, было выявлено 5 случаев биохимически подтвержденной акромегалии. Из них у 4 была подтверждена аденома гипофиза, по данным МРТ (магнитно-резонансной томографии) головного мозга, у 1 пациента изменений в структуре гипофиза выявлено не было [13]. Наши коллеги в 2020 г. также опубликовали результаты опроса пациентов с ассоциированными соматическими заболеваниями с помощью опросника Acroscreen, в котором приняли участие 1249 человек. Из них диагноз акромегалии удалось установить 9 участникам опроса [14].

В большинстве случаев причиной акромегалии является аденома гипофиза — соматотропинома. Гиперпролактинемия примерно в 25–39% случаев сопровождает избыток секреции гормона роста [15], а с другой — является распространенным эндокринным расстройством [16]. В российских клинических рекомендациях при выявлении акромегалии рекомендовано определение уровня пролактина [3]. При случайном выявлении опухоли гипофиза также есть рекомендации о рутинном скрининге акромегалии (при помощи определения уровня ИРФ-1) вне зависимости от наличия/отсутствия характерных симптомов [17]. Однако на сегодняшний день нет четкого понимания необходимости скрининга акромегалии у пациентов с опухолью гипофиза, диагностический путь которых начинается с гиперпролактинемии.

## ЦЕЛЬ ИССЛЕДОВАНИЯ

Целью исследования было определение доли пациентов с гиперпролактинемией и аденомой гипофиза, у которых исследовали уровень ИРФ-1 в условиях реальной клинической практики, а также выявление доли пациентов с акромегалией среди данной когорты.

## МАТЕРИАЛЫ И МЕТОДЫ

Исследование проводилось в клинико-диагностическом центре ГБУЗ МО МОНИКИ им. М.Ф. Владимирского в период с декабря 2019-го по декабрь 2022 гг.

Было проведено одноцентровое обсервационное одномоментное одновыборочное неконтролируемое исследование.

## Методы

На первом этапе исследования, по данным медицинских карт, путем сплошного включения наблюдений была проведена оценка доли пациентов с аденомой гипофиза и гиперпролактинемией, у которых исследовался уровень ИРФ-1.

На втором этапе исследования доступным для наблюдения пациентам без известных показателей ИРФ-1 проведено его определение.

Далее данные по частоте выявления акромегалии среди пациентов с известным уровнем ИРФ-1, полученные на первом этапе, были суммированы с данными на втором этапе.

При выявлении повышенного уровня ИРФ-1 было проведено исследование уровня СТГ в ходе теста с нагрузкой глюкозой. Диагноз акромегалии считался подтвержденным при выявленном повышении уровня ИРФ-1 выше половозрастной нормы в сочетании с отсутствием подавления уровня СТГ менее 1,0 нг/мл в ходе пробы с нагрузкой глюкозой.

Критерии включения:

Критерии исключения:

Для дополнительного анализа пациенты были разделены на две подгруппы в зависимости от первоначального повода для гормонального обследования: 1) инциденталома гипофиза, выявленная по результатам МРТ гипоталамо-гипофизарной области; 2) показания для определения уровня пролактина (жалобы на нагрубание и выделения из молочных/грудных желез, снижение либидо, репродуктивная дисфункция, избыточная масса тела, не связанная с алиментарными причинами), после подтверждения патологической гиперпролактинемии проводилась МРТ гипоталамо-гипофизарной области.

Анализ крови на ИРФ-1, СТГ выполнялся хемилюминесцентным иммунным методом на иммунохимическом анализаторе Unicel DxI 800, DBC (Diagnostics Biochem Canada), Canada.

МРТ с внутривенным контрастированием (Прохэнс) выполнялась на высокопольном аппарате со сверхсильной индукцией магнитного поля 3,0 Тл в режимах Т1ВИ, Т2ВИ, FLAIR до и после внутривенного введения контрастного препарата.

## Статистический анализ

С целью обработки непараметрических данных для определения середины множества чисел в изучаемой когорте использовалось определение медианы [минимальные значения; максимальные значения]. Данный параметр рассчитывался с помощью программы Microsoft Exсel.

Учитывая различия референсных значений уровней ИРФ-1 и пролактина в зависимости от пола и возраста, данные гормональные показатели представлены в виде процента превышения верхней границы референсных значений (ВГРЗ).

Для подсчета этого показателя использовалась следующая формула:

где У — уровень исследуемого гормона; ВГРЗу — верхняя граница референсных значений исследуемого гормона для данного пола и возраста пациента.

## Этическая экспертиза

Рассмотрение протокола настоящего исследования одобрено Независимым комитетом по этике при ГБУЗ МО МОНИКИ им. М.Ф. Владимирского, протокол заседания №6 от 17.06.2021 г.

РЕЗУЛЬТАТЫ

На первом этапе в исследование было включено 105 пациентов в возрасте от 18 до 85 лет, среди них — 79 женщин, 26 мужчин; с микроаденомой — 46 пациентов, с макроаденомой — 59. Медиана превышения уровня пролактина в дебюте заболевания составила 371,2 [ 38,6; 66050,1] %. Подгруппу с инциденталомами гипофиза составил 41 пациент, подгруппу с гиперпролактинемией — 64.

В целом из 105 пациентов уровень ИРФ-1 был определен в 41/105 (39%) случае; в том числе в подгруппе с инциденталомами гипофиза — в 22/41 (53,7%) случаях, в подгруппе с гиперпролактинемией — в 19/64 (29,7%) случаях.

На этом этапе исследования повышение уровня ИРФ-1 было зафиксировано у 7/41 человек (табл. 1, пациенты №1–7), по результатам проведенного дообследования акромегалия была подтверждена у 2 из них (оба из подгруппы инциденталом — табл. 1, пациенты №1 и 5).

**Table:** Таблица 1. Данные пациентов с гиперпролактинемией и аденомой гипофиза с повышенным уровнем ИРФ-1, в том числе с подтвержденной акромегалией Table 1. Data from patients with hyperprolactinemia and pituitary adenoma with elevated IGF-1 levels, including those with confirmed acromegaly Примечание. Подгруппа 1 — инциденталома гипофиза. Подгруппа 2 — гиперпролактинемия. ПРЛ — пролактин; ИРФ-1 — инсулиноподобный ростовой фактор 1 типа; СТГ — соматотропный гормон; ОГТТ — оральный глюкозотолерантный тест; 1 — возраст в момент определения ИРФ-1; 2 — до начала лечения заболевания; 3 — % превышения верхней границы половозрастных референсных значений; 4 — базальный уровень СТГ.

№	Возраст¹	Пол	Подгруппа	Объем аденомы², мм³	ПРЛ²,³, %	ИРФ-1³, %	СТГ⁴, нг/мл	Минимальное значение СТГ в ходе ОГТТ, нг/мл
1	30	ж	1	32	64,6	70,8	3,8	1,4
2	38	ж	1	486	545,1	6,2	4,9	0,9
3	71	ж	1	412,7	509,5	8,3	0,1	0,1
4	38	м	1	14 355	25 624	45,6	0,1	0,1
5	51	м	1	8748	121,4	181,3	5,9	2,6
6	60	ж	1	1428	259	14,6	0,46	0,1
7	68	ж	2	6510	11 750	115	3,76	0,3
8	25	ж	2	245	371,2	60,3	12,7	3,3
9	38	м	2	144	163,5	46,6	0,2	0,1
10	41	м	2	112	299,5	20,1	0,1	0,1
11	30	ж	2	1912	86,2	20,9	4,2	0,3

На втором этапе исследования уровень ИРФ-1 был определен еще у 53 пациентов с гиперпролактинемией и аденомой гипофиза. У 4 из них был зафиксирован повышенный уровень ИРФ-1 (табл. 1, пациенты №8–11), из которых у одного была подтверждена акромегалия (пациент №8).

В общей сложности уровень ИРФ-1 был определен у 94 больных с гиперпролактинемией и аденомами гипофиза в возрасте от 18 до 85 лет, среди них — 74 женщин, 20 мужчин; с микроаденомой — 42 пациента, с макроаденомой — 52. Медиана превышения уровня пролактина в дебюте заболевания составила 312,7 [ 38,6; 66050,1] %.

Подгруппа с инциденталомами включала 35 пациентов, подгруппа с гиперпролактинемией — 59 пациентов. Повышение уровня ИРФ-1 было зафиксировано у 11/94 пациентов (6 из подгруппы инциденталом, и 5 — из подгруппы гиперпролактинемии). Акромегалия была подтверждена у 3 из них (2 из подгруппы инциденталом, и 1 — из подгруппы гиперпролактинемии).

## ОБСУЖДЕНИЕ

Анализ данных медицинской документации 105 человек показал, что в реальной клинической практике исследование уровня ИРФ-1 было выполнено лишь у 41/105 (39%) пациента с гиперпролактинемией и аденомой гипофиза. Определение уровня ИРФ-1 проводится значительно чаще, если у пациента изначально выявлена опухоль гипофиза, а не гиперпролактинемия (53,7 против 29,7% соответственно). Тем не менее полученные данные говорят о том, что в реальной клинической практике не выполняются в полной мере рекомендации по исследованию уровня ИРФ-1 при случайно выявленной аденоме гипофиза [17]. Если же при диагностическом поиске складывается диагноз пролактиномы (гиперпролактинемия + выявленная опухоль гипофиза), скрининг акромегалии проводится еще реже.

На первом этапе исследования были выявлены 2 пациента с акромегалией до развития явных симптомов гиперсекреции гормона роста. Дополнительный скрининг уровня ИРФ-1 среди 53 пациентов помог диагностировать еще 1 случай акромегалии. Всего из 94 пациентов с аденомой гипофиза и гиперпролактинемией акромегалия была выявлена у троих (3,2%).

Andersen A., et al. в ходе 5-летнего наблюдения за 78 пациентами с пролактиномами выявили 3 случая акромегалии у пациентов без исходного повышения уровней ИРФ-1 и СТГ [18]. В исследовании Rosario P., еt al. из 121 пациента с пролактиномой на терапии каберголином диагноз акромегалии был установлен 5 пациентам без характерных клинических проявлений [19]. Таким образом, частота выявления акромегалии в этих исследованиях сопоставима с нашими данными.

Превышение верхней границы половозрастных референсных значений уровня ИРФ-1 у обследованных пациентов было отмечено в 11 случаях, и степень превышения варьировала от 6,2 до 181,3% (табл. 1). На изменение уровня ИРФ-1 могут повлиять различные факторы [20], однако среди наших пациентов были исключены нарушения функции щитовидной железы, почечная и печеночная недостаточность, женщины репродуктивного возраста не принимали оральные эстрогенсодержащие препараты, и у них была исключена беременность в момент обследования, мужчины не принимали препаратов тестостерона. У пациентов с подтвержденной акромегалией процент превышения ИРФ-1 составил более 50%. Возможно, определенная степень повышения уровня ИРФ-1 может послужить точкой отсечения при решении вопроса о целесообразности проведения нагрузочного теста с глюкозой для исключения акромегалии, необходимы дальнейшие исследования. Пациентам с любой степенью повышения уровня ИРФ-1 и физиологическим ответом СТГ на нагрузку глюкозой было проведено повторное тестирование, и уровень ИРФ-1 оказался в пределах половозрастных референсных значений.

У пациентки №7 отмечался значимо повышенный уровень ИРФ-1 — 115% (табл. 1). Однако в ходе теста с нагрузкой глюкозой было зафиксировано подавление СТГ менее 0,4 нг/мл, и при повторных тестированиях уровень ИРФ-1 не превышал половозрастные референсные значения.

У пациентки №2 (табл. 1) базальный уровень СТГ составил 4,9 нг/мл, минимальный СТГ в нагрузочном тесте — 0,9 нг/мл (в высокочувствительных тестах используется пороговое значение уровня СТГ менее 0,4 нг/мл). Однако превышение уровня ИРФ-1 составило лишь 6,2%, и при повторных исследованиях уровень ИРФ-1 не выходил за пределы половозрастной нормы.

Andersen A., et al. выдвинули гипотезу, что в аденомах гипофиза может отмечаться несинхронная гиперсекреция пролактина и гормона роста. Они предложили определять уровень ИРФ-1 не только в момент диагностики пролактиномы, но и ежегодно при динамическом наблюдении [18]. Наличие подавления уровня СТГ в ходе нагрузочного теста с глюкозой также не исключает в полной мере диагноза акромегалии [21][22]. Необходимо динамическое наблюдение за пациентами c любыми дискордантными показателями уровней ИРФ-1 и СТГ у пациентов с аденомами гипофиза, так как это может быть ранней стадией развивающейся акромегалии.

Как было показано в различных исследованиях с иммуногистохимическим анализом ткани удаленных опухолей гипофиза, повышение уровня пролактина у пациентов с акромегалией не коррелирует со способностью опухоли секретировать пролактин [23]. Поэтому в рамках заявленного дизайна исследования мы никак не можем судить о частоте соматопролактином или частоте гиперпролактинемии у пациентов с «чистыми» соматотропиномами.

У настоящего исследования есть ряд ограничений.

Количество включенных пациентов оказалось не столь велико, и для последующей экстраполяции данных на целевую популяцию требуются дальнейшие исследования.

На первом этапе исследования использовались данные амбулаторных карт, которые могли быть неполными.

Исследования уровня ИРФ-1 проводились в различных лабораториях, поэтому нельзя исключить вероятность лабораторной ошибки при получении повышенного уровня ИРФ-1. Этот факт также не позволяет сделать определенный вывод о точке отсечения % превышения ВГРЗ ИРФ-1, который можно было бы использовать для скрининга акромегалии.

Пациенты, у которых исследовался уровень ИРФ-1 на втором этапе, получали каберголин по поводу гиперпролактинемии. Результаты исследований о влиянии каберголина на уровень ИРФ-1 неоднозначны: есть данные о повышении уровня ИРФ-1 на фоне его приема [24], и есть данные об отсутствии влияния каберголина на уровень ИРФ-1 [25]. Из 53 пациентов, получавших каберголин, повышение уровня ИРФ-1 было отмечено в 5 случаях, что в большей степени поддерживает теорию об отсутствии влияния каберголина на уровень ИРФ-1 у лиц без акромегалии.

Кроме того, среди пациентов с гиперпролактинемией могли быть и пациенты с пролактиномами, и с симптоматической гиперпролактинемией вследствие сдавления ножки гипофиза. Без иммуногистохимической верификации типа опухоли или без клинического ответа на лечение каберголином мы не можем с уверенностью судить о типе гормональной активности опухоли гипофиза. Тем не менее, по данным некоторых исследований, можно сказать, что для гормонально-неактивных макроаденом гипофиза, которые сдавливают ножку гипофиза и являются причиной функциональной гиперпролактинемии, обычно характерен уровень пролактина менее 2000 мЕд/л [23] или менее 300% от ВГРЗ. В нашем исследовании было 43 пациента с превышением уровня пролактина менее 300%, из них 28 человек с микроаденомами гипофиза (уровень пролактина 128,4% [ 38,5; 299,5]) и 15 — с макроаденомами, которые могут быть условно отнесены к гормонально-неактивным опухолям гипофиза с функциональной гиперпролактинемией (уровень пролактина 137,2 [ 41,6; 299,3] %). Из пациентов с акромегалией, выявленной в ходе нашего исследования, у пациента №1 с микроаденомой (32 мм³) уровень пролактина превысил 64,6% ВГРЗ, и у пациента №8 с микроаденомой (245 мм³) уровень пролактина был более 300% ВГРЗ. У данных пациентов можно предположить, что аденома является пролактиномой, в то время как у пациента №5 была макроаденома гипофиза и повышение уровня пролактина (121,4% ВГРЗ), более характерное для сдавления ножки гипофиза. Таким образом, можно предположить, что при любом повышении уровня пролактина скрининг акромегалии будет оправдан.

## Направления дальнейших исследований

Как показывают упомянутые исследования, в том числе наше, гиперпролактинемия у пациента с аденомой гипофиза не исключает латентной гиперсекреции акромегалии. Это еще одно перспективное направление в раннем выявлении акромегалии. Проведенное нами исследование поддерживает предположение о том, что у пациентов с пролактиномами необходимо гормональное дообследование для исключения акромегалии.

## ЗАКЛЮЧЕНИЕ

Итоги нашего исследования показывают, что в реальной клинической практике у пациентов с аденомой гипофиза и гиперпролактинемией уровень ИРФ-1 исследуется лишь в 39% случаев. Даже если поводом для гормонального обследования стала случайно выявленная аденома гипофиза, определение уровня ИРФ-1 проводится только у 53% пациентов, несмотря на рекомендации определять уровень ИРФ-1 всем, независимо от клинических признаков акромегалии. Требуется активно применять существующие клинические рекомендации по гормональному обследованию инциденталом гипофиза для оптимизации ранней диагностики акромегалии.

Из 94 человек с гиперпролактинемией и аденомой гипофиза, у которых не было характерных клинических проявлений акромегалии, заболевание удалось выявить в 3 случаях (3,2%), что можно считать клинически значимым результатом. Учитывая распространенность сочетанного повышения пролактина и гормона роста у пациентов с аденомами гипофиза (на фоне сдавления ножки гипофиза соматотропиномой или совместной гиперсекрецией пролактина и гормона роста аденомой), мы считаем исследование уровня ИРФ-1 в качестве скрининга акромегалии у пациентов с гиперпролактинемией и аденомой гипофиза оправданным. Кроме того, результаты современных исследований говорят о том, что пациентам с пролактиномами можно рекомендовать ежегодное определение уровня ИРФ-1 в процессе динамического наблюдения [18][19].

## ДОПОЛНИТЕЛЬНАЯ ИНФОРМАЦИЯ

Источники финансирования. Работа выполнена по инициативе авторов без привлечения финансирования.

Конфликт интересов. Авторы декларируют отсутствие явных и потенциальных конфликтов интересов, связанных с содержанием настоящей статьи.

Участие авторов. Уханова Юлия Александровна, Иловайская Ирэна Адольфовна внесли существенный вклад в проведение данного исследования, написание статьи и внесение в рукопись существенной правки с целью повышения научной ценности статьи.

Все авторы одобрили финальную версию статьи перед публикацией, выразили согласие нести ответственность за все аспекты работы, подразумевающую надлежащее изучение и решение вопросов, связанных с точностью или добросовестностью любой части работы.
